# Visual outcome after small incision cataract surgery of patients in a Kenyan Hospital Contributors

**DOI:** 10.4314/ahs.v23i2.54

**Published:** 2023-06

**Authors:** Julius Kipkemboi Rono, Urvashni Nirghin

**Affiliations:** 1 School of public health and biomedical sciences and technology Department of optometry and vision Sciences, Masinde Muliro University of Science and Technology, Kakamega, Kenya; 2 Faculty of health sciences Department of Optometry, University of KwaZulu-Natal (Westville Campus), Durban,South Africa

**Keywords:** Small incision cataract surgery, visual outcome, refractive errors

## Abstract

**Background:**

The visual outcome and the effect of cataract surgery on existent and surgically induced refractive errors are a concern of interest that the eye care expert must deal with.

**Objectives:**

The aim of the study was to investigate the visual outcome after small incision cataract surgery of patients in a Kenyan hospital.

**Methods:**

A descriptive retrospective study covering 3 years (2015 to 2017). Total of 1104 files of patients aged > 50 years who had undergone small incision cataract surgery at Sabatia eye hospital were purposively selected. Information on demographics, clinical aspects and outcome was collected using a ministry of health structured form. Statistical package for social sciences version 24 was used to analyse the data.

**Results:**

Of the 1104 patients, 55% (n=606) were females. The mean age of patients was 70.6 ± 9.8 years. Most patients were from the hospital neighbourhood. Visual acuity improved by 38.9%, 28.9%, 19.8% and 11.5% in patients aged 50-59, 60-69, 70-79, and 80-89 years old, respectively.

**Conclusion:**

Small incision cataract surgery improved visual acuity in all patients' groups, but the outcome is influenced by age, refractive error particularly astigmatism.

## Introduction

Cataract is a multi-factorial condition 1. Pathogenesis of cataract development differs based on the underlying etiology. Cataracts are classified according to etiology, morphology, and maturity. There are Age-related cataracts, congenital cataracts 2, Secondary and Traumatic cataracts 3.

Cataracts are the leading cause of blindness worldwide, accounting for 51% of total blindness and 33% of total visual impairment 4. Many visually impaired individuals reside in developing countries 4. Blindness triggered by cataracts remains one of the biggest public health problems of our time resulting in economic and social hardships 5.

Four types of surgical intervention can be used to treat cataract. They are; Intracapsular cataract extraction (ICCE), Extracapsular cataract extraction (ECCE), Phacoemulsification (PE) and small incision cataract surgery (SICS). This study focused on the small incision cataract surgery, assessing the visual outcomes.

Apart from the patient's age, type of cataract, systemic conditions, and other ocular diseases, cataract extraction procedures affect the visual outcome. National and global research have proved Small Incision Cataract Surgery (SICS) a promising procedure with “good” visual outcomes obtained in more than 85 percent of cases with related complications occurring minimally 7. SICS is a commonly used low-cost surgical technique in developing countries for high-volume cataracts, with results being a “Good” visual outcome. It has the advantage of a self-sealing sutureless wound and also has advantage over other types like phacoemulsification, Extracapsular Cataract Extraction (ECCE) and Intracapsular Cataract extraction. SICSrequires identical ECCE tools, but has better-uncorrected vision 7, is cost- effective with small incision, fast and needs low-cost equipment and disposables. SICS is successful in more than 99% of cases operated. Its only disadvantages are large incision when compared to phaco-emulsification and only uses rigid lenses 10

Kenya occupies an area of 22,500 square miles in East Africa with a population of around 48 million 13. The incidence of blindness is reported at 0.7% with cataracts contributing 43%. In Kenya, eye units across hospitals adopt the same procedure - visual acuity using Snellen charts, same cataract examination methods, and patients that are operated on after one day, are rescheduled for clinic visits two weeks, four weeks, six weeks and after three months. The visual acuity and best-corrected visual acuity of the operated eye reported on the cataract audit form are measured throughout these appointments. “Poor” visual outcome is caused by several factors such as pre-existing eye and systemic diseases with ocular manifestations, surgical complications, spectacle failure to correct post-operative refractive error, and late postoperative complications 14.

Visual acuity (VA) is ideally adapted for assessing cataract surgery results, outcome or level of vision is therefore measurable following; the proportion of cataract operation that produces a given post-operative vision in the operated eye; the amount of recovered or restored blind eyes.

SICS is commonly associated with astigmatism, overcorrection and/or under correction, surgical, and post-operative complications, which ultimately affect the visual outcome 15, 7 14. study therefore investigates the visual outcome after small incision cataract surgeries in Kenya.

## Methods

A hospital-based retrospective (1^st^ January 2015 to 31^st^ December 2017), descriptive study carried out at Sabatia Eye Hospital (SEH) in Vihiga county, Kenya. A total of 1104 records were purposively selected out of 6423 records that had undergone cataract surgery in 2015, 2016 and 2017 who met all criteria needed to be part of the study. Patients 50 years and above were chosen, again those whose records were comprehensively done and who actually had the cataract extracted using small incision cataract surgery method was included 16, 17, 18, 19. Files that had incomplete entries were excluded.

Needed data was extracted from the records using the Standardized tool by the Kenya Ministry of health, division of ophthalmic services comprising of sections A to E. Section A, captures patients' demographics, Section B, the pre-operative ocular evaluation information, Section C, the practitioner's information. Section D captures the post-operative examination findings and Section E, captures likely causes of poor visual outcomes.

Data was analysed using SPSS version 24. Descriptive statistics was used in analyzing the pre- operative and post-operative visual outcomes. Visual findings are classified using the World Health Organization (WHO) classification where good vision is 6/6 to 6/18, borderline vision is <6/18 to 6/60, and poor vision is <6/60. The post-operative visual acuities on the 1^st^ day, 2^nd^ week, 6^th^ and 10^th^ week and above were recorded. Refraction was done at 8^th^ week post- operation.

A one-sample t-test was done to test whether the mean visual acuity for each post-operative period differed significantly from 0.3 with 0.3 being considered as the minimum visual acuity categorized as “Good”. The post-operative measurements or change scores on the 1^st^ day, 2^nd^ week, 6^th^ and 10^th^ week and above were compared using the dependent two-sample t-test 20. The hospital Ethics committee gave the needed permission for the study; consent was given by the hospital and approval gotten from the Kenyan Ministry of Health and the Biomedical Research and Ethics Committee – BE 270/17, of university of KwaZulu-Natal. The Helsinki declaration regarding obtaining information from patients' record and using same for studies was observed.

## Results

Of the 1104 records reviewed, there were more females 55% (n=606) (Table 1) with patients age ranging from 50 to 97 years old (70.6 ± 9.8) (Table 1). The mean age for males and females is shown in Table 1. More patients were from Kakamega County 17% (n=188), while Homabay County had the least number of patients 2.9% (n=32) (Table 1).

**Table 1:** Age, Gender and County of Residence of patients

Age group (years)	Male n (%)	Female n (%)	Total n (%)
50-59	83 (13.7)	83 (13.7)	166 (13.1)
60-69	177 (29.2)	177 (29.2)	334 (30.3)
70-79	203 (33.5)	203 (33.5)	406 (35.3)
80-89	127 (21.0)	127 (21.0)	254 (18.9)
90≥	16 (2.6)	16 (2.6)	32 (2.4)
Mean Age ±SD	70.3±9.5	70.8±10.0	70.6±9.8
	**County of residence of patients and gender**		
**County**	**Male n (%)**	**Female n (%)**	**Total n (%)**
Kakamega	87 (46.3)	101 (53.7)	188 (17.0)
Kisumu	75 (42.9)	100 (57.1)	175 (15.9)
Nandi	80 (48.5)	85 (51.5)	165 (15.0)
Uasin Gishu	51 (42.9)	68 (57.1)	119 (10.8)
Vihiga	50 (42.0)	69 (58.0)	119 (10.8)
Bungoma	17 (48.6)	18 (51.4)	35 (3.2)
Busia	19 (45.2)	23 (54.8)	42 (3.8)
Homa Bay	17 (53.1)	15 (46.9)	32 (2.9)
Siaya	44 (42.7)	59 (57.3)	103 (9.3)
Others	69 (54.8)	57 (45.2)	126 (11.4)
**Total n (%)**	**498 (46)**	**606 (54)**	**1104 (100)**

*Descriptive statistics, Data presented as Percentages (frequencies) (n=1104)*

**Figure 1:**
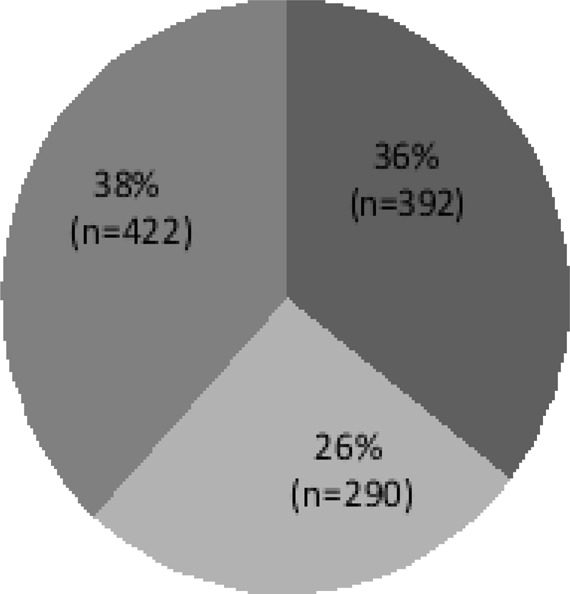
Distribution of patients that underwent SICS at SEH (2015, 2016 and 2017) Descriptive statistics, Data presented as Percentages (frequencies) (n=1104)

There was an uneven distribution of patients between the periods under review (2015, 2016 and 2017) as shown in Figure 1.

### Comparison of VA outcomes with the different age-groups and over time

The “in-subject” impact revealed that there were substantial variations in VA between respondents in age ranges of 50 to 59 years and 80 to 89 years (p<0.001).

The first VA assessment (pre-operative to the fifth assessment (10^th^ week post-operative) showed significant VA changes over time, by age groups and for time/age groups, but there was no significant VA change for gender (Table 3).

**Table 3:** VA outcomes among Age and between Genders over time

Source	Type III Sum of Squares	df	Mean Square	F	*p-value*	Partial Eta Squared
	**VA outcomes among age-groups over time**					
Time (1^st^ to 5^th^ assessment (Greenhouse-**Geisser)**	0.119	1.643	0.073	0.105	0.030	0.300
Age Groups	0.342	1.000	0.342	0.183	**0.001**	0.001
(80 – 89; 50 – 59; 60 – 69)						
	**VA outcomes between gender over time**					
Time	0.119	1.643	0.073	0.105	0.030	0.300
(Greenhouse-Geisser) Gender	0.480	1.000	0.480	0.256	0.614	0.002

Repeated Measure ANOVA from converted z-scores – 1^st^ assessment to 5th assessment statistically significant p<0.005

Comparison between uncorrected pre-operative and post-operative VA with follow-up time periods for SICS Based on WHO classification of “Poor”, “Borderline” and “Good” VA, there were more “Poor” VA pre-operatively, which reduced on the first day post-operative. Those categorized as “Good”, remained “Good” with their VA. See Table 4. Changes among those who had borderline improved from 12.3% (n=136) pre-operative to 29.3% (n=323) on the 1st day post- operative. At 6 weeks' post-operative, 27.2% (n=148) patients had good uncorrected VA, compared to 61.3% (n=228) who were in the same category after correction or ‘best corrected’. 10 weeks' post-operative, the category of good vision improved from 33.8% (n=79) for uncorrected to 69.4% (n=114) for best-corrected VA. The proportion of patients reporting poor vision at 10 weeks was still higher of 14.1% (n=33) than the recommended WHO rate of less than 5% 21.

**Table 4:** Uncorrected Pre-operative and Post-operative VA with follow-up time periods for SICS

WHO Categories	Pre-op VA n (%)	1^st^ Day Post-op VA n (%)	2 Weeks Post-op VA n (%)	2 Weeks Post-op Pinhole VA n (%)	6 Weeks Post-op VA n (%)	6 Weeks Post-op BCVA n (%)	≥10 Weeks Post-op VA n (%)	≥10 Weeks Post-op BCVA n (%)
Good	10(0.9)	254 (23.1)	495 (45.2)	291 (45.5)	148 (27.2)	228 (61.3)	79 (33.8)	114
								(69.4)
Borderline	136(12.3)	323 (29.3)	476 (43.4)	262 (41.0)	281 (51.6)	109 (29.3)	122 (52.1)	41 (25.2)
Poor	958(86.8)	525 (47.6)	125 (11.4)	86 (13.5)	116 (21.2)	35 (9.4)	33 (14.1)	8 (4.9)
Total	**1104(100)**	**1102 (100)**	**1096 (100)**	**639 (100)**	**545 (100)**	**372 (100)**	**234 (100)**	**163 (100)**

*Data presented asfrequencies (percentages) (n= 1104)*

### Comparison of post-operative mean best-corrected VA between 2nd to 10th week

There was significant improvement in post-operative VA after correction between the 2^nd^ and 6^th^ week (p = 0.003), 6^th^ and 10^th^ week (p = 0.004), and 2^nd^ and 10^th^ week (p = 0.001).

### Pre-operative and post-operative refractive errors

Refractive errors are determined at 6^th^ week and 10^th^ week post SICS at SEH, out of the sample of 1104, 63% (n = 695) presented with no pre-operative refractive errors, which thereafter increased to 84.7% (n=935) post-operatively. There was a decrease in the various refractive error classifications from the 37% who had pre-operative RE. Of those with uncorrected pre- operative refractive errors (37%), simple myopic astigmatism decreased from 1.3% (n=14) pre- operatively to 0.5% (n=6) post-operatively. The highest proportion of the patients presented with pre-operative compound hyperopic astigmatism of 18.1% (n=200) which decreased to 0.3% (n=4) post-operatively See Table 6.

**Table 6:** Pre-operative and Post-Operative Refractive Errors

Type of Refractive Error (Pre-operative and Post-operative)	Pre-operative n (%)	Postoperative n (%)	*Rho (p*-Value)
No refractive error	695 (63.0)	935 (84.7)	0.023
Compound myopic astigmatism	109 (9.9)	120 (10.9)	0.137
Compound hyperopia astigmatism	200 (18.1)	4 (0.3)	**0.001**
Simple hyperopia astigmatism	16 (1.4)	2 (0.2)	0.298
Mixed astigmatism	70 (6.3)	37 (3.4)	0.053
Simple myopic astigmatism	14 (1.3)	6 (0.5)	0.439
**Total n (%)**	**1104 (100)**	**1104 (100)**	

## Discussions

Majority of the patients in the study and in all age group intervals were females, however, the findings showed no significant difference between male and female. A study in China by Song *et al.* (2018) and in Sweden by Zetterber and Celojevic (2014), showed a similar trend. As there was a higher cataract incidence among females than males, the reduced estrogen levels in post-menopausal years among females could be the attributing factor 22, 23. Khanna *et al.*, (2007), their study showed that estrogen is believed to have many anti-aging properties that may justify the longer lifespan of women 22.

Even though the study identified patients undergoing SICS at SEH from as early as 50 years old, the mean age was 70.6 ± 9.8 years. The majority of the patients were in the<=““ span=”” style=“font-family: “Times New Roman”; font-size: 12pt; letter-spacing: 0.2pt;”>age range of 70 to 79 years. This is an indication that cataract is a condition largely related to age. Studies have shown similar results, in WHO world bulletin Negrel et al., (1995), and in China by Song *et al*, (2018) 24, 23, 3. Apart from age being a risk factor to cataract, there are other risk factors associated with advanced age, for example, diabetes and hypertension 3.

Most of the patients in this study came from Kakamega County (17%) which is explained by the proximity of SEH to the localities. According to a 2009 census, Kakamega county is the second-most populated county in Kenya, with a population of approximately over 1,100,987 (Kenya National Bureau of Statistics, 2009 census). The results have a clear indication that the closer to the health facility, the more the attendance of the patients, therefore, cataract services should be brought closer to patients, implying an eye hospital should be set up in every county. In 2017, a greater number of patients underwent SICs at SEH, followed by the year of 2015, with the least being attended to in 2016. A general pattern was noticed and that of a decline in visit from 2015 to 2016 followed by an increase from 2016 to 2017 even between genders. This was a clear indication of an uneven distribution of the number of patients who visited the hospital during the selected time of the study. The drop in patients' number in 2015 to 2016 could have caused an accumulative effect in the year 2017.

### Pre- and post-operative VA following SICS at SEH

The marked change in VA over time could be the result of the reduction in inflammation over time following SICS 25. Also, SICS has been shown to havebetter prognosis compared to large incision cataract surgery 9. The prognosis is better in small incision cataract surgery because there are fewer complications compared to large incisions.

There was a significant improvement in VA in all the age groups on first-day post-operative (p<0.001), with the greatest improvement reported among the age range of 50-59 years old. Age is a risk factor for cataract formation, hence the older the patient, the greater the risk of cataract development and progression 3. In addition to the relationship between age and cataracts, additional systemic risk factors such as diabetes and hypertension, could have led to a minimal improvement in VA in the older patients. The findings above concur with other studies 3. Raman (2010) stated that co-morbidities were significantly associated with poor visual outcome (p<0.001) 26.

A comparison in pre- and post-operative VA by gender revealed that the changes in VA between males and females were comparable over the 10-week period. There was a notable improvement in VA among males and females on day one and second week, followed by a decline in the sixth week (p<0.023), and again an improvement in the 10th week (p<0.002), but there was no significant difference in the distribution of VA by gender. Albulena (2018) managed to study sex related differences in vision and males out-performed females on fifteen common measures of visual perception 27. This was not possible in this study because the research was focused only on visual outcome.

### Comparison between uncorrected pre-operative and post-operative VA with follow up time period for SICS

There were changes in uncorrected VA pre-operative and post-operative as well as after the best correction post-operatively. WHO, (2007) categorizes the outcome of cataract surgery into three groups: good, borderline and poor 21. WHO further recommends that a good, uncorrected VA post-operatively should be at least 80%, while the poor outcome should be less than 5% 21. This study was consistent with WHO (2007) as results showed that after cataract surgery, there was improvement of poor vision from 86.8% pre-operatively to 47.6% on the first-day post-operatively 21. Data from this study therefore confirms that SICS improved VA on patients. This is supported by Sonron (2015) in their retrospective study in an Eastern Regional Health Authority Hospital of Trinidad and Tobago, in which 67% of the patients had improved VA following cataract surgery 28. As the main aim of cataract surgery is to improve the vision of patients, the data collected in this study supports the conclusion that cataract surgery improves the VA of patients in SEH. Olawoye (2011) reported similar results in their study 29. Despite the positive outcome in a study by Kongsap (2011), the pre-operative VA was lower compared to this study 9.

Cataract surgery using small incision has been established with good visual outcome. Therewas a significant improvement in VA of cataract surgery patients. Similarly, patients who wereon borderline vision improved from 12.3% to 29.3% on the first post-operative day. Agreeing with findings by Stock (2015), where a retrospective analysis of 4924 cataract surgeries was conducted from the Veterans Healthcare Administration Ophthalmic Surgical Outcomes Data Project (OSOD) 30. The results also showed improvement from uncorrected to best corrected VA (33.8% to 69.4%) due to the fact that the number of population size reduced during subsequent follow ups. This is a common observation and several studies have noted the same 31. The proportion of patients reporting poor vision at 10 weeks still remained higher 33 (14.1%) than the recommended WHO rate of less than 5% 21. Possible reason could be due to the complications resulting from sequel. There was a significant gain in VA on the first day (p<0.003), second week (p<0.002) and the tenth week (p<0.001). Overall, the change was evident between the first week and tenth-week post-operative (p<0.001). On the contrary, between the second and the sixth week, there was no significant improvement (p=0.165). Despite recording an insignificant gain in VA between the 2nd week post-operative and 6th week post-operative, there was a significant ncrease in VA at week 10 post-operative. This can be explained from the delayed recovery of the surgery at week 2 and week 6. Several studies have reported similar results that show an improvement in VA after cataract surgery. Masum and colleagues (2015) reported again in uncorrected VA patients after cataract surgery 6. A study done in Oman displayed an excellent grade of vision gain in relation to pre- operative VA status. This was reported on 64.6% cases of cataract surgery. Of all the studied cases, good VA gain was 12.1%, while poor VA was 9.6%. The results from these studies suggest that catarct is a major cause of reversible loss of vision with significant recovery following cataract surgery.

There was a significant improvement in best-corrected VA after SICS between day one post- operative and week 10 post-operative. This was an indication that refractive errors are the major cause of poor VA before and after surgical interventions. A study similar to the current one indicated an improvement of VA after refractive correction eight weeks post-operative 29.

### Pre- and post-operative refractive errors

The presence of refractive errors in an operated eye could be due to a pre-existing refractive error before surgery or a refractive error induced during the surgery. Modern microsurgical approaches, modern IOL tools, innovative biometry methods and improved IOL power measurement methods render it possible for most cataract patients to restore high-quality vision (National Institute for Health and Care Excellence, 2017). The key problem to prevent a refractive shock after cataract surgery is the precision of the IOL measurement and the option of the correct biometric method for each case 8. This study finding revealed that refractiveerrors increased to 84.7% post-operatively in the category of no refractive error pre-operatively. Possible reason for this is that the density of the cataract made it impossible to determine the exact number of cases with refractive errors pre-operatively. The decrease of simple myopic astigmatism from 1.3% pre-operatively to 0.5% post-operatively could have been due to the correct estimation of the intraocular lens inserted together with the small incision done. Studies have reported mean myopic differences in spherical equal refraction of 0.70 D from 1 day postoperative to 2 months 11. Compound myopic astigmatism, on the other hand, increased from pre-operative 9.9% to post-operative 10.9%. Astigmatism induced by the surgical procedure could be thecause of this kind of refractive error after cataract surgery. Furthermore, refractive error may be affected by operational factors such as surgical differences in the scale and central location of the capsulorhexis, which can affect the final position of the IOL within the bag and are dependent on the skill of the surgeon 12.

## Conclusion

From the study, more women were recorded to have reported poor vision due to cataracts which was rescued using small incision cataract surgery. Arguably, if men had more record, there could have been a difference to this fact and this leaves room for further gender exploration of this title in a possible further study. This could be explained by the fact that women form a majority of the population that seeks health intervention at SEH.

A comparison of VA before and after SICS at SEH indicates that there was a significant improvement in visual outcome from day one to the 10th week post-operatively. This indicates that SICS is an effective management for poor vision as a result of cataract formation. It was also found that the younger age group had better visual outcomes post-operatively compared to the elderly.

Refractive errors with co-morbidities like diabetes, hypertension, ARMDs, and glaucoma were the major causes of poor visual outcome after SICS. Patients with pre-existing refractive errors reported poor VA compared. Moreover, compound myopic astigmatism was the most common refractive error after SICS resulting in poor vision.

## Figures and Tables

**Table 2:** VA outcomes with different age and over time

Age in Years	Age in Years	Mean	Standard Error	p-Value
50-59	60-69	1.875	0.644	0.180
	80-89	14.300	1.031	0.000^*^
60-69	50-59	-1.875	0.644	0.180
	80-89	12.425	0.775	0.000^*^
80-89	50-59	-14.300	1.031	0.000^*^
	60-69	-12.425	0.775	0.000^*^

*Bonferroni Post-hoc test.*

* *Statistically significant results p<0.001*

**Table 5:** Post-operative Mean Best Corrected VA

Operative status	n (%)	Mean in (meters)	SD	Rho (p-Value)
Post-operative BCVA 2 weeks versus BCVA 6 weeks	343 (54.2)	-0.07	0.23	**0.003**
Post-operative BCVA 6 weeks versus BCVA 10 weeks	159 (25.1)	-0.03	0.20	**0.004**
Post-operative BCVA 2 weeks versus BCVA 10 weeks	131 (20.7)	-0.10	0.20	**0.001**
